# Validation of a Farsi version of the Eating Disorder Examination Questionnaire (F-EDE-Q) in adolescents and university students from Iran

**DOI:** 10.1186/s40337-023-00830-y

**Published:** 2023-06-30

**Authors:** Reza N. Sahlan, Jessica F. Saunders, Patrycja Klimek-Johnson, Alexandra D. Convertino, Jason M. Lavender, Ellen E. Fitzsimmons-Craft, Jason M. Nagata

**Affiliations:** 1 Department of Counseling, School, and Educational Psychology, Graduate School of Education, University at Buffalo-SUNY, Buffalo, NY USA; 2 Psychology Convening Group, Ramapo College of New Jersey, Mahwah, USA; 3 San Francisco VA Medical Center, University of California, San Francisco, USA; 4 University of California San Diego Joint Doctoral Program in Clinical Psychology, San Diego State University, San Diego, CA USA; 5 Military Cardiovascular Outcomes Research (MiCOR) Program, Department of Medicine, Uniformed Services University of the Health Sciences, Bethesda, MD USA; 6 The Metis Foundation, San Antonio, TX USA; 7 Department of Psychiatry, Washington University in St. Louis, St. Louis, USA; 8 Department of Pediatrics, University of California, San Francisco, CA USA

**Keywords:** Eating Disorder Examination-Questionnaire, F-EDE-Q, Adolescents, University, Assessment, Iran

## Abstract

**Background:**

Although the Farsi version of the Eating Disorder Examination-Questionnaire (F-EDE-Q) is frequently utilized to assess disordered eating in Iran, its factor structure, reliability, and validity have not been investigated in Iranian samples, which is the aim of the current investigation.

**Method:**

Using convenience sampling, this study recruited 1112 adolescents and 637 university students to complete disordered eating and mental health-focused questionnaires, including the F-EDE-Q.

**Results:**

Confirmatory factor analyses of the 22 attitudinal items in the F-EDE-Q indicated that a brief seven-item, three-factor model (i.e., Dietary Restraint, Shape/Weight Overvaluation, Body Dissatisfaction with Shape and Weight) was the only factor structure that fit the data well for either sample. This brief version of the F-EDE-Q was invariant across gender, body weight, and age. Adolescent and university participants with higher weight reported higher average scores on each of the three subscales. Subscale scores showed good internal consistency reliability in the two samples. Further, supporting convergent validity, subscales were significantly associated with measures of body image-related preoccupation and bulimia symptoms, as well as measures of other theoretically related constructs including depressive symptoms and self-esteem.

**Conclusion:**

Findings suggest that this brief, validated measure will enable researchers and clinical providers to appropriately assess disordered eating symptoms in adolescent and young adult Farsi-speaking populations.

## Abstract (highlights)

The Eating Disorder Examination-Questionnaire (EDE-Q) is a questionnaire that assesses disordered eating. This study assessed whether the Farsi-language version of the EDE-Q is acceptable to use in Iranian adolescent and university populations. Participants in the study answered the items on the F-EDE-Q and other questionnaires about mental health. Results indicated that a short version of the F-EDE-Q including seven items and three subscales was the only version that was appropriate to use with both adolescents and university students. This brief version of the F-EDE-Q measured eating disorder symptoms similarly across weight status, gender, and age. Adolescent and university samples with higher weight had higher average scores on each of the three subscales.

## Background

Recent studies have shown high rates of disordered eating, including binge eating, vomiting, laxative misuse, and excessive exercise among Iranian adolescents and university students [1–5]. These symptoms appear to be similar across genders [1–3], and clinically significant levels of disordered eating are comparable between university students (19.4%) and adolescents (17.3%) [3]. Surprisingly, a cross-cultural study by Sahlan et al. [6] found that Iranian university women reported binge eating and purging at the same rate as their counterparts in the United States (US), challenging the notion of eating disorders as exclusively Western phenomena. Previous research has also identified common risk factors for eating disorders in Iran, including Western sociocultural influences and social comparison [6–9]. Given these findings, it is crucial to evaluate existing measures for assessing disordered eating symptoms in Iranian individuals to facilitate reliable and valid eating disorder symptom assessment by researchers and clinicians.

One of the most widely used measures of disordered eating is the Eating Disorder Examination-Questionnaire (EDE-Q; [10]). The EDE-Q was originally developed to assess disordered eating symptoms based on a theoretical, rather than empirical approach [11]. Older versions of the EDE-Q included 36 items; however, the current version of the measure is comprised of 28 items, 22 of which contribute to the global score and the four subscale scores: Restraint, Eating Concern, Weight Concern, and Shape Concern. Several studies have found poor psychometric support for the original four-factor structure of the EDE-Q in adult samples from the US [12–16], United Kingdom (UK; [17]), and Norway [18]. Thus, researchers have proposed and examined alternative factor structures.

Two alternative models of the EDE-Q have gained strong support across various adult samples [12, 18]. The first alternative is a 22-item, four-factor model (Dietary Restraint, Preoccupation and Restriction, Weight and Shape Concerns, Eating Shame) which was supported in a community sample of Norwegian women [18], university students from the US [16], and sexual minority men and women from the US [14]. Research has also supported measurement invariance based on gender for this alternative version, including in samples of university men and women and sexual minority men and women from the US [14, 16]. The second alternative is a brief seven-item, three-factor model (Dietary Restraint, Shape/Weight Overvaluation, Body Dissatisfaction with Shape and Weight) by Grilo et al.’s [12] which was supported in bariatric surgery candidates [12], sexual minority men and women from the US [14], university students from the US [13, 15, 16], and university students from the UK [17]. This model has also demonstrated measurement invariance based on gender, body mass index (BMI), and ethnicity in university men and women from the US [13, 15, 16], sexual minority men and women from the US [14], and university men and women from the UK [17].

Multiple factor structures for the EDE-Q also have been evaluated in adolescent samples. For example, a study by Kass et al. [19] in children with overweight or obesity from the US found support for Grilo et al.’s [12] brief seven-item, three-factor model (described above). Further, in a sample of adolescent boys and girls from the US, White et al. [20] found support for a 22-item, three-factor model (Shape and Weight Concerns, Restriction, Preoccupation and Eating Concern). A 22-item, two-factor model (Restraint and Eating/Shape/Weight Concern) has also demonstrated good model fit and was found to be invariant across gender among mixed rural and urban adolescents in Mexico [21]. Another study among Indian adolescents supported a two-factor model (Preoccupation and Control, Weight and Shape Concerns) with 15 and 18 items in girls and boys, respectively [22], which was largely invariant across gender. Thus, support has been found for several alternative models of the EDE-Q in various adolescent samples.

Prior studies have utilized the Farsi version of the EDE-Q (F-EDE-Q; [1, 2]). However, the factor structure of the F-EDE-Q and measurement invariance based on gender and BMI status have not been examined among Iranian adolescents and university students. Previous studies have focused on the global score of the F-EDE-Q [1, 9], with limited examination of the original four subscales [2]. Concerningly, certain F-EDE-Q subscales have shown questionable internal consistency reliability (e.g., α = 0.61 for Shape Concern among men, α = 0.69 for Eating Concern among women) [2]. However, a comprehensive evaluation of alternative F-EDE-Q score factor structures and psychometric properties in Iranian samples has not yet been conducted. Notably, there are inconsistencies in the evaluated alternative models of the EDE-Q between child/adolescent and young adult samples from Western countries [12, 20]. Disordered eating symptoms are common in adolescents and university students in Iran (e.g., clinical threshold on the EDE-Q [17–19%], binge eating [20–27%], purging [2–7%]; [1–3) and university students had higher body dissatisfaction than adolescents [23]. However, it is unclear whether the F-EDE-Q performs similarly in samples reflecting distinct stages of the lifespan (i.e., adolescents and young adults). Thus, examining the measurement invariance of the F-EDE-Q by age group is warranted. To the best of our knowledge, there is only one study in which an alternative measure of disordered eating (i.e., Farsi version of the Eating Pathology Symptoms Inventory; F-EPSI; [23]) similarly across age groups (i.e., adolescents vs. university students); however, these results would not be generalizable to the F-EDE-Q.

### Current study

Utilizing samples of Iranian adolescents and university students, the aims of this study were to (a) identify the best fitting factor structure of the F-EDE-Q in each sample; (b) evaluate measurement invariance across theoretically relevant subgroups (i.e., gender [13, 16, 17], BMI status [13], and age [24, 25]), and conduct group comparisons if appropriate; and (c) examine the convergent validity of the best-fitting model of the F-EDE-Q in each of the samples. Study 1 focused on a sample of Iranian adolescent boys and girls, and Study 2 focused on Iranian adult university men and women. Study 3 used the combined sample to explore measurement invariance across age groups.

### Study 1

We first assessed which model—of the previously published models with adolescents—was the best fitting in the sample of Iranian adolescents: (a): White et al.’s [20] 22-item, three-factor model, (b): Penelo et al.’s [21] 22-item, two-factor model, (c): Grilo et al.’s [12] brief seven-item, three-factor model (supported by Kass et al. [19] , or (d): Lewis-Smith et al.’s [22] two-factor model with 15 and 18 items for girls and boys, respectively. This approach, rather than a more exploratory data-driven approach (i.e., exploratory factor analysis), was undertaken to ensure comparability and facilitate meaningful comparisons with previous research conducted in different populations, including adolescents and university students. These models have been extensively studied, and their psychometric properties and factor structure have been well-documented in multiple studies [12, 19, 21, 22]. Upon selecting the best fitting model, we examined measurement invariance by gender and BMI status prior to comparing group-level mean differences [26]. We hypothesized that girls and adolescents with higher BMI would report higher scores on the F-EDE-Q versus boys and those with a lower BMI, respectively. We expected that the identified subscales of the best-fitting model would show at least adequate internal consistency reliability. Additionally, consistent with findings from Western and non-Western samples [1, 13, 17, 27, 28], we hypothesized that the convergent validity of the F-EDE-Q scores would be supported based on significant positive associations with scores on measures of body image-related preoccupation and bulimia symptoms (moderate to large in effect size), as well as significant positive associations with the score on a measure of depressive symptoms (small to moderate in effect size) and significant negative associations with the score on a measure of self-esteem (small to moderate in effect size).

### Study 2

In Study 2, we first assessed which model—of the previously published models with university samples—was the best fit among Iranian university students (a): Friborg et al.’s [18] 22-item, four-factor model or (b): Grilo et al.’s [12] brief seven-item, three-factor model. Similar to Study 1, we examined the measurement invariance of the best-fitting model. In line with other studies, we expected that the F-EDE-Q would be invariant across gender [13–18] and BMI status. We hypothesized that participants with higher (versus lower) BMI would report higher mean scores on the F-EDE-Q, and that women and men would have comparable scores, in line with prior research with this population [2]. We expected that the identified subscales of the best-fitting model would show at least adequate internal consistency. Further, in accordance with previous studies [1, 13, 17, 27–29], we hypothesized that the convergent validity of the F-EDE-Q scores would be supported based on significant positive associations with scores on a measure of body image-related preoccupation (moderate to large in effect size), as well as significant positive associations with the score on a measure of depressive symptoms (small to moderate in effect size) and significant negative associations with the score on a measure of self-esteem (small to moderate in effect size).

### Study 3

Finally, we combined samples from Study 1 and 2 to evaluate measurement invariance of the best-fitting model of the F-EDE-Q across age groups (i.e., adolescence versus early adulthood).

### Study 1: evaluating factor structure, measurement invariance, and convergent validity of the F-EDE-Q in Iranian adolescents

The aim of Study 1 was to use confirmatory factor analysis (CFA) to examine alternative F-EDE-Q factor structures based on those supported for the EDE-Q in previous studies [12, 13, 19–22], as well as to evaluate measurement invariance (across gender and BMI status), internal consistency reliability, and convergent validity in a sample of Iranian adolescents.

## Method

### Participants and procedure

Sample 1 (*N* = 1112, 54.6% girls) was also utilized in Sahlan et al.’s study [3], but the current investigation has unique aims and novel primary analyses. Participants (*N* = 1,112) were adolescent boys (*n* = 504) and girls (*n* = 607) aged 12–19 years. The mean age was 15.35 (*SD* = 1.43) years for boys and 15.71 (*SD* = 1.70) years for girls. Boys’ standardized BMI (zBMI) ranged from − 2.77 to 3.78 (*M* = 0.01, *SD* = 1.05), while girls' ranged from − 2.51 to 3.67 (*M* = -0.05, *SD* = 0.95). The sample was recruited from 19 schools (9 boys'’ schools and 10 girls' schools) and 154 classes across Tehran, Tabriz, Kurdistan, and Rasht. Only 27.1% of eligible students had the option to participate due to instructor participation being optional. Adolescents completed a paper-and-pencil survey in the presence of research staff without compensation. The order of the scales was counterbalanced, and anonymity was ensured to protect confidentiality. Research procedures were approved by school and regional administrators, parental consent was obtained, and adolescents provided assent. The study received approval from the institutional review board of a large Iranian university.

## Measures

### Demographics questionnaire

Participants reported their age, gender, height, and weight (used to derive BMI; kg/m^2^; [30]) on a study-specific questionnaire. Aligned with World Health Organization guidelines for children and adolescents aged 5–19 years, we converted BMI to age- and sex-specific zBMI scores [31]; these scores were used to categorize adolescents into groups based on BMI status: (1) underweight: z-score <  − 2 standard deviations (*SD*), (2) average weight: between ≥  − 2*SD* and ≤  + 1*SD*, (3) overweight: >  + 1*SD*, or (4) obesity >  + 2*SD*). Due to small sample sizes for groups with underweight and obesity, and in line with other studies among adolescents in Iran [32, 33], we dichotomized the BMI status variable by collapsing the underweight (*n* = 7 [0.6%]) and average weight (*n* = 909 [82.2%]) (zBMI score < 1) groups together, as well as collapsing the overweight (*n* = 139 [12.6%]) and obesity (*n* = 42 [3.8%]) (zBMI score ≥ 1) groups together.

### Farsi-Eating Disorder Examination-Questionnaire (F-EDE-Q)

The F-EDE-Q [2] was used to assess disordered eating symptoms over the past 28 days. Participants rate 22 items on a seven-point scale, with higher scores reflecting more severe disordered eating symptoms. In the original structure of the measure, four subscales are calculated based on the average of the respective items, and a global score can be calculated as the average of those subscale scores.

### Farsi-Preoccupation of Eating, and Weight or Shape (F-PEWS)

The F-PEWS [1] was used to assess cognitive preoccupation with eating, weight, and shape. The scale requires a respondent to rate the percentage of the day they spend thinking about eating or weight and shape. Participants completed six items rated on a seven-point scale ranging from 0 (*not at all*) to 6 (*extremely*). This measure was included to evaluate the convergent validity of the F-EDE-Q subscales [1, 29]. Internal consistency values for this and all subsequent measures appear in Table 4.

### Farsi-Eating Disorder Inventory-2-Bulimia Scale (F-EDI-2-B)

The bulimia scale of the F-EDI-2 [34] was used to assess bulimia symptoms. Participants rate items on a seven-point scale ranging from 1 (*never*) to 7 (*always*), with higher scores indicating greater bulimic symptomatology. This measure was included to evaluate the convergent validity of the F-EDE-Q subscales [17, 28].

### Farsi-Rosenberg Self-Esteem Scale (F-RSES)

The F-RSES [35] was used to assess global self-esteem. The scale includes 10 items rated on a four-point scale ranging from 1 (*strongly disagree*) to 4 (*strongly agree*), with higher scores reflecting higher self-esteem. This measure was included to evaluate the convergent validity of the F-EDE-Q subscales, given the theoretical and empirical relevance of self-esteem in relation to disordered eating [13, 27].

### Farsi-Beck depression inventory (F-BDI-II)

The F-BDI-II [36] was used to assess depressive symptoms. The scale includes 21 items which are rated on a four-point scale ranging from 0 (*did not apply to me at all*) to 3 (*applied to me very much, or most of the time*); higher scores reflect greater depressive symptomatology. This measure was included to evaluate the convergent validity of the F-EDE-Q subscales, given the commonly overlapping nature of disordered eating and depressive symptoms [13, 17].

## Statistical analyses

One participant did not provide demographic information (e.g., age, gender, weight, height) and was excluded from the analyses. Additionally, several adolescent boys did not complete the F-EDE-Q (*n* = 6, 1.2%), F-PEWS (*n* = 6, 1.2%), F-EDI-2-B (*n* = 6, 1.2%), F-RSES (*n* = 8, 1.6%), and F-BDI-II (*n* = 7, 1.4%). With respect to demographic information, 3–4 boys (0.6–0.8%) and 2–3 girls (0.3–0.5%) did not provide their weight and/or height to calculate BMI. If participants began a scale, they completed that scale in its entirety with no missing data within scales. In line with previous research [32, 33], this small amount of missing data was handled via listwise deletion, as it was not possible to impute full measures [37]. The final sample size was 1,106 adolescents.

Data were examined for skewness and kurtosis prior to analysis. Consistent with previous studies [20–22], we used CFA to examine multiple possible factor structures of the F-EDE-Q, including White et al.’s [20] 22-item, three-factor model (i.e., Shape and Weight Concerns, Restriction, Preoccupation and Eating Concern), Penelo et al.’s [21] 22-item, two-factor model (Restraint and Eating/Shape/Weight Concern Subscales), and Lewis-Smith et al.’s [22] two-factor model with 15 or 18 items (Preoccupation and Control, Weight and Shape Concerns). As suggested in the literature [38, 39], we assessed model fit using the Root Mean Square Error of Approximation (RMSEA; < 0.08 indicates a good model fit) and its 90% confidence interval [40], the Comparative Fit Index (CFI; > 0.95 indicates a good model fit), and the Tucker–Lewis Index (TLI; > 0.95 indicates a good model fit).

For the best fitting model, we examined measurement invariance by gender and zBMI [41] using multi-group confirmatory factor analysis (MGCFA), testing the models both constrained and unconstrained by gender and separately by zBMI status (i.e., <  + 1SD and ≥  + 1SD). Factor loadings were first constrained to be equal across groups and then allowed to be freely estimated across groups in each instance to establish equal form and subsequently test for metric invariance. We then tested a further constrained model in which factor loadings and intercepts were constrained to be equal across groups and then allowed to be freely estimated across groups to assess scalar invariance. Because the chi-square (*χ*^*2*^) statistic is sensitive to large samples, metric equivalence was determined if the fit statistics (CFI, TLI, RMSEA) change by approximately 0.01 or less from when the model paths are constrained to when they are freely estimated [42]. We used AMOS 26.0 to conduct CFA and MGCFA.

Internal consistency reliability for the F-EDE-Q subscales scores was evaluated using Cronbach’s alpha (values α ≥ 0.70 considered acceptable; [43]) and McDonald’s omega (ω ≥ 0.70 considered acceptable; [44, 45]). However, the recommended reliability coefficient for two-item subscales is the Spearman-Brown coefficient (*ρ*), as it is considered less biased [46]. Pearson’s correlations were used to examine associations between the F-EDE-Q subscales scores and to evaluate convergent validity (i.e., correlations with F-PEWS, F-EDI-2-Bulimia scale, F-BDI, and F-RSES). Correlation coefficients of 0.10, 0.30, and 0.50 were considered to be small, medium, and large, respectively [47]. We also calculated the composite reliability coefficient as well as the Average Variance Extracted (AVE) as evidence of convergent validity. The composite reliability coefficient is an indicator of the shared variance among the observed values that each subscale of the F-EDE-Q is comprised of [48]. AVE is a measure of the amount of variance that is captured by a construct in relation to the amount of variance due to measurement error [48]. Fornell and Larcker [48] recommend an AVE above 0.50 and a composite reliability score above 0.60. Upon establishing measurement invariance across gender and BMI status, independent samples *t*-tests (equal variances) and Welch’s *t*-test (unequal variances) were used to examine gender and BMI status (zBMI <  + 1SD vs. zBMI ≥  + 1SD) differences on the F-EDE-Q subscales scores among adolescents. SPSS 25.0 and JASP 0.14 were used for these analyses.

## Results

### Factor structure and measurement invariance

**Fig. 1:**
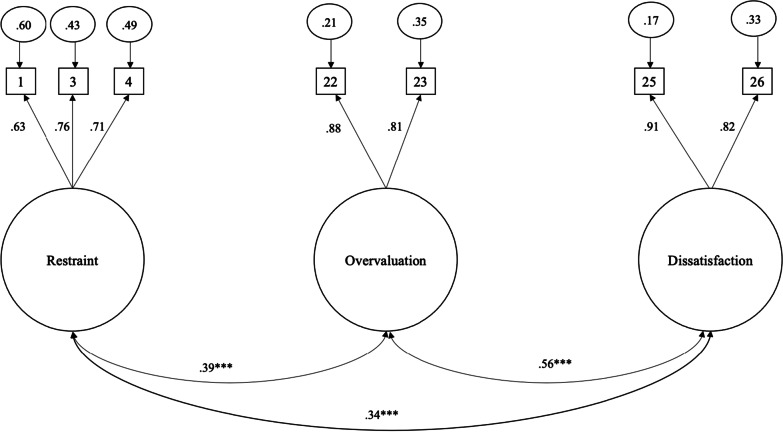
Standardized Factor Loadings for Iranian Adolescents in Study 1. *Note*. Restraint = Restraint over eating (item 1), Food avoidance (item 3), and Dietary Rules (item 4). Overvaluation = Importance of weight (item 22), Importance of shape (item 23). Dissatisfaction = Dissatisfaction with weight (item 25), Dissatisfaction with shape (item 26). ^***^ *p* < .0.001

Data were evaluated for normality and then analyzed using maximum likelihood estimation. Assessment of univariate indices of kurtosis and skewness revealed skewness and kurtosis values below the absolute value of 2, falling within the normal range [39]. Mardia’s test of normality was significant for both skewness (*z* = 200.52, *p* < 0.001) and kurtosis (*z* = 32.49, *p* < 0.001); the subsequent analyses were bootstrapped to address the non-normality. The only model that fit the data well was Grilo et al.’s [12] brief seven-item, three-factor model (see Table 1 for the fit statistics for each other alternative model tested), χ^2^ (11) = 37.73, *p* < 0.001; RMSEA = 0.04, 90% CI [0.03, 0.06], *p* = 0.70; CFI = 0.99, TLI = 0.98. All items loaded strongly and significantly onto their respective factor, with factor loadings ranging from 0.71 to 0.91. As this was the only acceptable model, it was used for subsequent examination of measurement invariance, group-level differences, reliability, and validity. The model demonstrated metric invariance by both gender (Δχ^2^ [4] = 1.93, *p* = 0.75; ΔRMSEA = 0.004; ΔTLI = 0.005; ΔCFI = 0.001) and BMI status (Δχ^2^ [4] = 3.10, *p* = 0.54; ΔRMSEA = 0.002; ΔTLI = 0.005; ΔCFI = 0.001). The model also demonstrated scalar invariance by gender (Δχ^2^ [7] = 36.37, *p* < 0.001; ΔRMSEA = 0.005; ΔTLI = 0.008; ΔCFI = 0.009) but not by BMI status (Δχ^2^ [7] = 104.82, *p* < 0.001; ΔRMSEA = 0.03; ΔTLI = 0.06; ΔCFI = 0.04). The standardized factor loadings and intercorrelations between subscales for the entire adolescent sample are depicted in Fig. 1.

**Table 1:** Summary of Models Tested in Iranian Adolescents

Model	χ^2^	RMSEA	TLI	CFI
White et al. [20]	χ^2^ (186) = 28140.59, *p* < 0.001	0.11 [90% CI 0.10, 0.12]	0.69	0.75
Penelo et al. [21]	χ^2^ (208) = 31280.473, *p* < 0.001	0.11 [90% CI 0.10, 0.12]	0.68	0.73
Lewis-Smith et al. girls [22]	χ^2^ (89) = 6820.13, *p* < 0.001	0.11 [90% CI 0.10, 0.12]	0.81	0.84
Lewis-Smith et al. boys [22]	χ^2^ (134) = 6450.37, *p* < 0.001	0.09 [90% CI 0.08, 0.09]	0.82	0.86
Grilo et al. [12, 13]	χ^2^ (11) = 370.73, *p* < 0.001	0.04 [90% CI 0.03, 0.06]	0.98	0.99

### Internal consistency, composite reliability, and intercorrelations

As summarized in Table 2, F-EDE-Q Dietary Restraint, Shape/Weight Overvaluation, and Body Dissatisfaction with Shape and Weight demonstrated good internal consistency based on Cronbach’s alphas, Spearman-Brown coefficients for two-item subscales, and McDonald’s omegas. The seven-item scale also demonstrated strong Composite Reliability (CR = 0.92), above the cut-off recommended by Fornell and Larcker [48]. The three subscales had small-to-moderate, positive intercorrelations.

**Table 2:** Internal consistency and intercorrelations among Iranian adolescents (study 1) and university students (study 2)

Brief Seven-F-EDE-Q Subscales	Cronbach’s ɑ or Spearman-Brown coefficient	McDonald’s ω	1	2	3
*Adolescents*					
1. Dietary Restraint	0.73 (Cronbach’s ɑ)	0.73	–		
2. Shape/Weight Overvaluation	0.83 (Spearman-Brown)	0.83	0.34**	–	
3. Body Dissatisfaction with Shape and Weight	0.85 (Spearman-Brown)	0.85	0.37**	0.51**	–
*University students*					
1. Dietary Restraint	0.78 (Cronbach’s ɑ)	0.78	–		
2. Shape/Weight Overvaluation	0.85 (Spearman-Brown)	0.85	0.31**	–	
3. Body Dissatisfaction with Shape and Weight	0.83 (Spearman-Brown)	0.83	0.28**	0.47**	–

*Note*. ^**^ *p* < .001

### Gender and BMI status differences

Girls showed significantly higher scores than boys on the F-EDE-Q Dietary Restraint and Body Dissatisfaction with Shape and Weight subscales, and no gender differences emerged for Shape/Weight Overvaluation (see Table 3). Additionally, adolescents with a higher zBMI had significantly higher scores on each of the three F-EDE-Q subscales.

**Table 3:** Descriptive data and t-tests for Iranian adolescents (study 1), university students (study 2), and the combined sample (study 3)

	Boys (*n* = 498)	Girls (*n* = 607)	*t*	*p*	Cohen’s *d*^*a*^
	*M (SD)*	*M (SD)*			
*Study 1: adolescents*					
Dietary restraint^b^	1.23 (1.52)	1.50 (1.64)	2.83	0.005	0.17
Shape/weight overvaluation^b^	1.86 (1.95)	2.01 (1.91)	1.25	0.211	0.08
Body dissatisfaction with shape and weight	1.75 (1.85)	2.25 (2.07)	4.20	0.0001	0.25

	zBMI < 1 (*n* = 916)	zBMI ≥ 1 (*n* = 181)			
Dietary restraint	1.22 (1.50)	2.22 (1.79)	7.92	0.0001	0.61
Shape/weight overvaluation	1.79 (1.85)	2.77 (2.12)	6.30	0.0001	0.49
Body dissatisfaction with shape and weight	1.76 (1.82)	3.44 (2.19)	10.95	0.0001	0.83

	Men (*n* = 253)	Women (*n* = 384)			
*Study 2: university students*					
Dietary Restraint^b^	1.14 (1.52)	1.27 (1.57)	1.07	0.285	0.08
Shape/Weight Overvaluation^b^	2.03 (1.85)	2.08 (1.91)	0.30	0.767	0.03
Body dissatisfaction with shape and weight	2.02 (1.65)	2.29 (1.95)	1.85	0.064	0.15

	BMI < 25 (*n* = 525)	BMI ≥ 25 (*n* = 111)			
Dietary restraint	0.99 (1.39)	2.26 (1.84)	8.17	0.0001	0.78
Shape/weight overvaluation^b^	1.94 (1.84)	2.62 (2.0)	3.27	0.001	0.35
Body dissatisfaction with shape and weight	1.93 (1.71)	3.40 (1.94)	8.0	0.0001	0.80

	Adolescents (*n* = 1106)	University students (*n* = 637)			
*Study 3: combined sample of adolescents and university students*					
Dietary restraint^b^	1.39 (1.59)	1.22 (1.56)	2.13	0.033	0.11
Shape/weight overvaluation^b^	1.95 (1.94)	2.07 (1.89)	1.26	0.209	0.06
Body dissatisfaction with shape and weight	2.03 (1.99)	2.19 (1.84)	1.66	0.096	0.08

^a^Effect size (i.e., small [0.0–0.2], medium [0.2–0.5], large [≥ 0.8])

^b^Levene’s test for equality of variance was violated and unequal variances were assumed

zBMI score < 1 = Low-to-Average weight. zBMI score ≥ 1 = Higher weight. BMI score < 25 = Low-to-Average weight. BMI score ≥ 25 = Higher weight

### Convergent validity

F-EDE-Q Dietary Restraint, Shape/Weight Overvaluation, and Body Dissatisfaction with Shape and Weight demonstrated significant, positive associations (small-to-large in size) with bulimia symptoms, preoccupation with eating, weight or shape, and depressive symptoms. The F-EDE-Q subscales were also significantly negatively associated with self-esteem. All correlations are presented in Table 4. Additionally, the seven-item scale demonstrated an AVE of 0.63, above the cut-off recommended by Fornell and Larcker [48].

**Table 4:** Internal consistency and pearson correlations among Iranian adolescents in study 1

	Cronbach’s α	McDonald’s ω	F-EDE-Q Dietary Restraint	F-EDE-Q Shape/Weight Overvaluation	F-EDE-Q Body Dissatisfaction with Shape and Weight
Eating preoccupation	0.77	0.84	0.30**	0.29**	0.38**
Shape/weight Preoccupation	0.81	0.88	0.30**	0.38**	0.56**
Overall Preoccupation	0.87	0.90	0.32**	0.37**	0.51**
Bulimia Symptoms	0.82	0.83	0.17**	0.25**	0.27**
Self-esteem	0.85	0.86	− 0.06*	− 0.19**	− 0.32**
Depressive Symptoms	0.92	0.92	0.07*	0.27**	0.32**
zBMI	–	–	0.30**	0.24**	0.33**

F-EDE-Q = Brief seven-item, three-factor Farsi-Eating Disorder Examination-Questionnaire. Eating Preoccupation and Shape/Weight Preoccupation = Two subscales of the Farsi Preoccupation of Eating and Weight or Shape (F-PEWS); Overall Preoccupation reflects the total score. Bulimia = Subscale of the Farsi Eating Disorder Inventory-Second edition (EDI-2). Self-esteem = Farsi Rosenberg Self-Esteem Scale. Depression = Farsi Beck Depression Inventory-II. zBMI = Standardized Body Mass Index

*p < 0.05

**p < 0.001

### Study 2: evaluating factor structure, measurement invariance, and convergent validity of the F-EDE-Q in Iranian university students

The aim of Study 2 was to examine F-EDE-Q factor structure using CFA (four-factor model, three-factor model; [12, 18]), as well as evaluating measurement invariance (across gender and BMI status) and convergent validity of the F-EDE-Q in a sample of Iranian university students.

## Method

### Participants and procedure

Sample 2 (*N* = 637, 60.3% women) was also utilized in Sahlan et al. [2], but the current project has unique aims and novel primary analyses. The sample consisted of young men (*n* = 253) and women (*n* = 384) recruited from the University of Tabriz and the University of Shiraz in Iran. Participants' age range was 18–54 years, with men having a mean age of 21.86 (*SD* = 3.16) years and women having a mean age of 21.91 (*SD* = 3.89) years. Self-reported BMI ranged from 15.85 to 39.18 (*M* = 22.88, *SD* = 3.50) for men and 15.57 to 36.73 (*M* = 21.74, *SD* = 3.42) for women. Potential participants from various departments were approached on campus and provided information about the study. Those who agreed to participate completed the paper–pencil scales in the presence of research staff without compensation. Scale order was counterbalanced, and anonymity was ensured for confidentiality. The study received approval from the institutional review board of a large Iranian university.

## Measures

### Demographic questionnaire

Participants reported their age, gender, height, and weight on a study-specific questionnaire. BMI (kg/m^2^) was subsequently calculated, and consistent with recommendations from the National Heart, Lung, and Blood Institute (National Institutes of Health [NIH]; [49]) and the World Health Organization ([WHO]; [50]). BMI was used to classify participants into categories of underweight (BMI < 18.50), average weight (BMI 18.50–24.99), overweight (BMI 25–29.99), and obesity (BMI ≥ 30). Based on these criteria, *n* = 85 (13.3%) participants were classified as having underweight,* n* = 440 (69.1%) participants were classified as having average weight, *n* = 92 (14.4%) participants were classified as having overweight, and *n* = 19 (3.0%) participants were classified as having obesity. To ensure adequate power and in line with previous studies [32, 33], the BMI status variable was dichotomized by combined the underweight (*n* = 85 [13.3%]) and average weight (*n* = 440 [69.1%]) groups (*n* = 525 [82.40%]), as well as combining the overweight (*n* = 92 [14.4%]) and obesity (*n* = 19 [0.3%]) groups (*n* = 111 [17.40%]) to examine further measurement invariance across BMI status.

### Additional measures

Consistent with Study 1, the F-EDE-Q, F-PEWS, F-RSES, and F-BDI-II were also administered to the adult university men and women in Study 2. We did not administer the F-EDI-2 to university samples.

### Statistical analyses

One participant (0.4%) did not provide weight and height to calculate BMI. There were no missing data on any of the scales administered. Consistent with previous studies [13, 14, 16, 18], we examined the factor structure of the F-EDE-Q subscales scores through CFA to evaluate Grilo et al.’s [12] three-factor model (i.e., Dietary Restraint, Shape/Weight Overvaluation, Body Dissatisfaction with Shape and Weight) and Friborg et al.’s [18] four factor-model (Dietary Restraint, Preoccupation and Restriction, Shape and Weight Concerns, Eating Shame). Using the best fitting models, we examined measurement invariance by gender and BMI status. Group-level and validity analyses were conducted similarly to those described in Study 1.

## Results

### Factor structure and measurement invariance

**Fig. 2:**
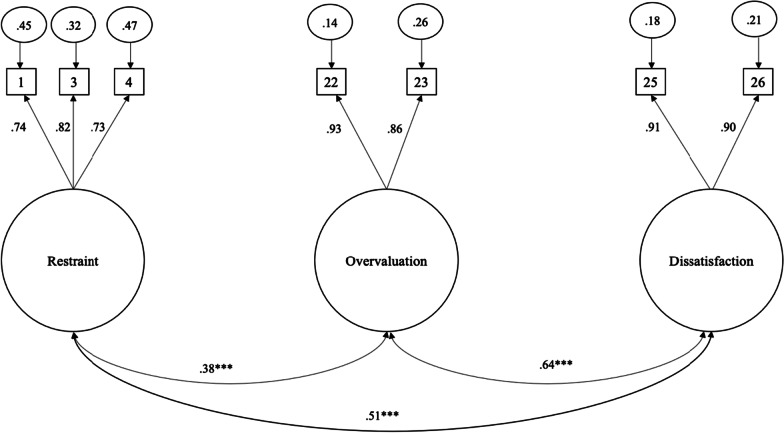
Standardized Factor Loadings for Iranian University Students in Study 2. *Note*. Restraint = Restraint over eating (item 1), Food avoidance (item 3), and Dietary Rules (item 4). Overvaluation = Importance of weight (item 22), Importance of shape (item 23). Dissatisfaction = Dissatisfaction with weight (item 25), Dissatisfaction with shape (item 26). ^***^ *p* < .0.001

Data were evaluated for normality and then analyzed using maximum likelihood estimation. Assessment of univariate indices of kurtosis and skewness revealed skewness and kurtosis values below the absolute value of 2. Mardia’s test of normality was significant for both skewness (*z* = 157.22, *p* < 0.001) and kurtosis (*z* = 32.23, *p* < 0.001); the subsequent analyses were bootstrapped to address the non-normality. The only model tested that fit the data well was Grilo et al.’s [12]  three-factor model with seven items: χ^2^ [11] = 21.32, *p* = 0.03; RMSEA = 0.05, 90% CI [0.02, 0.08], *p* = 0.47; CFI = 0.99, TLI = 0.99. Friborg et al.’s [18] four-factor model fit the data poorly: χ^2^ (204) = 2634.42, *p* < 0.001; RMSEA = 0.14, 90% CI [0.13, 0.14], *p* < 0.001; CFI = 0.68, TLI = 0.64. In Grilo et al.’s [12] three-factor model, all items loaded strongly and significantly onto their respective factor, with factor loadings ranging from 0.73 to 0.93. As this was the only acceptable model, this was the model we proceeded with for measurement invariance, group-level differences, and validity analyses. The model demonstrated metric measurement invariance by both gender (Δχ^2^ [4] = 2.73, *p* = 0.60; ΔRMSEA = 0.003; ΔTLI = 0.003; ΔCFI = 0.001) and BMI status (Δχ^2^ [4] = 3.64, *p* = 0.46; ΔRMSEA = 0.003; ΔTLI = 0.003; ΔCFI < 0.001). The model also demonstrated scalar invariance by gender (Δχ^2^ [7] = 12.85, *p* = 0.08; ΔRMSEA = 0.000; ΔTLI = 0.003; ΔCFI = 0.002) but not by BMI status (Δχ2 [7] = 72.22, *p* < 0.001; ΔRMSEA = 0.032; ΔTLI = 0.046; ΔCFI = 0.039). The standardized factor loadings and intercorrelations between subscales for the entire university sample are depicted in Fig. 2.

### Internal consistency, composite reliability, and intercorrelations

As summarized in Table 2, F-EDE-Q Dietary Restraint, Shape/Weight Overvaluation, and Body Dissatisfaction with Shape and Weight demonstrated good internal consistency based on Cronbach’s alphas, Spearman-Brown coefficients for two-item subscales, and McDonald’s omega. The seven-item scale also demonstrated strong Composite Reliability (CR = 0.94), above the cut-off recommended by Fornell and Larcker [48]. The subscales were positively associated with moderate-to-large correlations.

### Gender differences and BMI status

As summarized in Table 3, no significant gender differences emerged for F-EDE-Q Dietary Restraint, Shape/Weight Overvaluation, and Body Dissatisfaction with Shape and Weight. However, university students with higher BMI showed significantly higher scores on the three F-EDE-Q subscales.

### Convergent validity

The F-EDE-Q Shape/Weight Overvaluation and Body Dissatisfaction with Shape and Weight subscales were significantly, positively associated with preoccupation with eating, weight or shape and depressive symptoms as well as negatively associated with self-esteem (see Table 5). Dietary Restraint had a significant positive association with preoccupation with eating, shape or weight, but was not significantly associated with depressive symptoms or self-esteem. Correlations ranged from small to large in size (see Table 5). The seven-item scale also demonstrated an AVE of 0.71, above the cut-off recommended by Fornell and Larcker [48].

**Table 5:** Internal consistency and pearson correlations among Iranian university students in study 2

	Cronbach’s α	McDonald’s ω	F-EDE-Q Dietary restraint	F-EDE-Q shape/weight overvaluation	F-EDE-Q body dissatisfaction with shape and weight
Eating preoccupation	0.82	0.90	0.40**	0.38**	0.45**
Shape/weight preoccupation	0.85	0.93	0.39**	0.44**	0.50**
Overall preoccupation	0.91	0.94	0.41**	0.42**	0.49**
Self-esteem	0.87	0.86	− 0.05	− 0.22**	− 0.27**
Depressive symptoms	0.91	0.92	0.04	0.21**	0.25**
BMI	–	–	0.41**	0.18**	0.31**

F-EDE-Q = Brief seven-item, three-factor Farsi-Eating Disorder Examination-Questionnaire. Eating Preoccupation and Shape/Weight Preoccupation = Two subscales of the Farsi Preoccupation of Eating and Weight or Shape (F-PEWS); Overall Preoccupation reflects the total score. Self-esteem = Farsi Rosenberg Self-Esteem Scale. Depression = Farsi Beck Depression Inventory-II

*BMI* body mass Index

**p < .001

### Study 3: measurement invariance of the brief seven-item, three-factor F-EDE-Q across age groups

The F-EDE-Q based on Grilo et al.’s [12] brief seven-item, three-factor model showed adequate fit in the adolescent and adult university samples in Study 1 and Study 2. Therefore, the aim of Study 3 was to examine measurement invariance in this model by age group in a combined sample.

## Method

Please refer to procedures and measures for Study 1 and Study 2.

### Statistical analyses

Consistent with approaches taken in Study 1 and Study 2, MGCFA was used to examine measurement invariance by age group in the combined adolescent and university samples. Independent sample *t*-tests (equal variances) and Welch’s *t*-test (unequal variances) were subsequently used to examine differences on the F-EDE-Q subscales scores in adolescents versus university students.

## Results

To assess metric invariance, the item factor loadings were first estimated freely and then constrained to be equal across groups. Grilo et al.’s [12] brief three-factor model demonstrated metric invariance by age, Δχ^2^ (4) = 2.13, *p* = 0.70; ΔRMSEA = 0.003; ΔTLI = 0.004; ΔCFI =  < 0.001 and scalar invariance by age, Δχ^2^ (7) = 1.69, *p* = 0.79; ΔRMSEA = 0.007; ΔTLI = 0.04; ΔCFI = 0.001. This finding suggests it is appropriate to use the same brief three-factor model with both Iranian adolescents and young adults. As summarized in Table 3, adolescents endorsed significantly higher scores on F-EDE-Q Dietary Restraint, but no other significant group differences between adolescents and university students were found.

## General discussion

In the current study, a brief seven-item, three-factor version of the F-EDE-Q (i.e., Dietary Restraint, Shape/Weight Overvaluation, Body Dissatisfaction with Shape and Weight; [12]) was found to be the only factor structure to demonstrate good fit in samples of Iranian adolescents and university students. Evidence also supported internal consistency of the F-EDE-Q subscales corresponding to this three-factor version in both samples, as well as supporting convergent validity. In line with previous studies [13, 14, 16, 24, 25], this model was found to be invariant based on gender and BMI status in both samples, as well as across age groups in the combined sample. These findings therefore suggest that the same latent constructs are being measured by the three F-EDE-Q subscales across the specified groups, allowing for valid group comparisons based on the specified demographic characteristics. Taken together, these findings support the brief seven-item, three-factor version of F-EDE-Q as a reliable and valid instrument of assessing disordered eating symptoms in Iranian youth and young adults.

Each of the three subscales of this brief version of the F-EDE-Q exhibited adequate to good internal consistency. This is an important finding, particularly given that previous research found questionable internal consistency for certain subscales based on the original four-factor structure of the measure [2]. Moreover, the current study found that there was evidence supporting convergent validity of the brief seven-item, three-factor F-EDE-Q. Specifically, the subscales were significantly associated with body image-related preoccupation and bulimia symptoms, which was in line with previous studies in Western samples [17, 51]. Also, consistent with previous studies in Western societies [17, 27, 52–54], Shape/Weight Overvaluation and Body Dissatisfaction with Shape and Weight showed acceptable, but weaker than expected, associations with self-esteem and depressive symptoms in the adolescent sample. Dietary Restraint also had a small but significant association with self-esteem and depression in adolescents, which mirrors findings from a previous study in the UK [17]. However, the strength of the associations of the three subscales with the measures of depressive symptoms and self-esteem were generally lower than those reported in previous studies [17, 27, 52–54].

Consistent with prior studies using the EDE-Q [20, 55], adolescents in the current sample were found to endorse greater Dietary Restraint compared to the university students, although no significant group differences were found for Shape/Weight Overvaluation or Body Dissatisfaction with Shape and Weight. This may suggest that certain factors related to risk for dietary restraint and restrictive eating behaviors are especially salient at younger ages. For example, sociocultural influences on dieting behaviors have been found to be stronger for adolescents than for university students [56].

With regard to gender differences, we found that adolescent girls reported higher scores on Dietary Restraint and Body Dissatisfaction with Shape and Weight than adolescent boys, which aligns with previous studies in Western adolescent samples [57–60]. Notably, there were no gender differences in Shape/Weight Overvaluation in either sample, suggesting that salience of body shape and weight is similar across male and female youth and young adults. Taken together, these findings may suggest that the severity of cognitive (body image concerns) and behavioral (dietary restraint) features of eating disorders may differ by age and gender among Iranian adolescents and young adults. Thus, researchers and clinicians should consider the potential differences among these populations when assessing for disordered eating symptoms.

The current findings also contribute to the literature on disordered eating symptoms in relation to weight status. Consistent with previous studies in Western and Iranian samples [32, 61], both adolescents and university students with higher weight endorsed higher scores on all three F-EDE-Q subscales, indicating that greater body weight is broadly associated with more disordered eating symptoms. Notably, contrary to previous studies in Western samples [17, 54], zBMI/BMI were both found to be correlated with Shape/Weight Overvaluation in the current samples. This finding suggests the particular salience of body weight in relation to disordered eating symptoms among Iranian adolescents and university students, and aligns with recent prior work from Iran [32].

### Strengths and limitations

This study is the first to empirically examine alternative factor structures of the F-EDE-Q, along with reliability, validity, and measurement invariance in Iranian adolescents and university students. Strengths include the large sample sizes, the inclusion of both boys/men and girls/women, and the use of confirmatory factor analytic approaches for evaluating multiple factor structures and measurement invariance. However, some limitations of the current study should be noted. First, this study focused solely on the binary categorization of male and female, overlooking other gender identities. Given the prevalence of disordered eating symptoms across diverse gender identities [62], future research should comprehensively investigate these variations. Second, the F-EDE-Q was only administered at one time point; thus, test–retest reliability could not be examined. Third, associations of the brief seven-item, three-factor F-EDE-Q with depressive symptoms and self-esteem were small, suggesting the need for future examination of convergent validity with other constructs. Fourth, although the brief version of the F-EDE-Q was broadly supported in the current samples of adolescents and university students, the findings may not be generalizable to other groups, such as children [63], older adult populations, and patients with an eating disorder diagnosis in Iran. Accordingly, examining the brief three-factor model in other age groups and clinical samples would be a necessary next step in ensuring the F-EDE-Q is a valid and reliable assessment of disordered eating behaviors for all Farsi speaking individuals. Further, BMI was calculated based on self-reported height and weight. Although this approach has certain limitations, self-reported body weight has been shown to correlate highly with actual weight (e.g., *r*s ~ 0.90; [64, 65]), suggesting that it is a suitable alternative when height/weight cannot be measured directly. Fifth, measures were not available for evaluating the discriminant validity, or other potentially relevant psychometric properties (e.g., predictive validity), of the brief version of the F-EDE-Q in the current samples. Finally, it should be noted that this brief version of the F-EDE-Q only measures two general features of disordered eating symptoms (i.e., dietary restraint and body image-related concerns). As such, future research investigating the psychometric properties of Farsi-language versions of other more comprehensive and multi-faceted eating disorder symptom questionnaires will be needed [23, 66].

## Conclusion

Eating disorder symptoms are prevalent among Iranian adolescents and young adults, challenging the perception that they are solely Western phenomena. However, psychometric examinations of self-report measures of disordered eating in Iran have been largely absent. In this study, we examined different factor structures of the Farsi-language EDE-Q in Iranian adolescents and university students. Only a brief seven-item, three-factor model (Dietary Restraint, Shape/Weight Overvaluation, Body Dissatisfaction with Shape and Weight) demonstrated good fit in both samples, aligning with Western literature. This version of the F-EDE-Q was invariant across gender, body weight status, and age groups. The subscales showed satisfactory internal consistency and were significantly associated with body image concerns and bulimia symptoms, supporting convergent validity. However, associations with depressive symptoms and self-esteem were weaker. Overall, a brief version of the F-EDE-Q based on Grilo’s [12] model shows promise as a self-report tool for assessing certain disordered eating symptoms in research and clinical settings in Iran.

## Data Availability

The data that support the findings of this study are available on request from the first author.

## Abbreviations

EDE-Q: Eating Disorder Examination-Questionnaire
F-EDE-Q: Farsi version of the EDE-Q
F-EPSI: Farsi version of the Eating Pathology Symptoms Inventory
F-PEWS: Farsi-Preoccupation of Eating, and Weight or Shape
F-EDI-2-B: Farsi-Eating Disorder Inventory-2-Bulimia scale
F-RSES: Farsi-Rosenberg Self-Esteem Scale
F-BDI-II: Farsi-Beck Depression Inventory
RMSEA: Root Mean Square Error of Approximation
CFI: Comparative Fit Index
TLI: Tucker–Lewis Index
CFA: Confirmatory Factor Analysis
MGCFA: Multi-Group Confirmatory Factor Analysis
AVE: Average Variance Extracted
CR: Composite Reliability
NIH: National Institutes of Health
WHO: World Health Organization
BMI: Body Mass Index
zBMI: Standardized BMI
SD: Standard Deviations
